# Identification of the Potential chi-miR-148a-5p-PTGS2 Regulatory Axis Mediating TNF and IL-17 Signaling Pathways in Caprine Bronchial Epithelial Cells Challenged with Klebsiella Pneumoniae

**DOI:** 10.3390/vetsci13090953

**Published:** 2026-09-12

**Authors:** Yimei Chen, Zhenxing Gong, Hui Jing, Hao Duan, Hejie Qian, Tengjiao Li, Shihua Niu, Lili Wu, Ziying Wang, Mengqi Zhang, Yiwen Cheng, Churiga Man, Qiaoling Chen, Li Du, Si Chen, Hongyan Gao, Fengyang Wang

**Affiliations:** Hainan Key Laboratory of Tropical Animal Reproduction & Breeding and Epidemic Disease Research, School of Animal Science and Veterinary Medicine, Hainan University, Haikou 570228, China; 9202508730@alumni.hainanu.edu.cn (Y.C.); zhenxinggong@yeah.net (Z.G.);

**Keywords:** *Klebsiella pneumoniae*, mRNA-miRNA sequencing, TNF signaling pathway, IL-17 signaling pathway

## Abstract

*Klebsiella pneumoniae* (*K. pneumoniae*) belongs to the genus *Klebsiella* of the *Enterobacteriaceae* family. In goats, *K. pneumoniae* infection can result in respiratory diseases such as pneumonia and bronchopneumonia. In this study, caprine bronchial epithelial cells were infected with *K. pneumoniae*, and multi-omics integration analysis was conducted to investigate the immune defense mechanisms triggered by the infection. Enrichment analysis of differentially expressed genes (DEGs) using the KEGG database revealed that immune-related signaling pathways involving TNF and IL-17 were among the top 20 most enriched. Based on the TNF and IL-17 signaling pathways, ten immune pathway-related genes, including *CCL20*, *CXCL1*, *TRAF4*, *FOS*, *IL6*, *PTGS2*, *TNF*, *CXCL8*, *TNFAIP3*, and *JUN*, were selected for RT-qPCR validation. In addition, four miRNAs targeting genes involved in the above pathways, namely chi-miR-148a-5p, miR-1285-z, miR-16-x, and miR-6119-x, were verified by RT-qPCR. The expression trends of chi-miR-148a-5p and miR-1285-z were consistent with microRNA sequencing results. Furthermore, gain- and loss-of-function experiments indicated that chi-miR-148a-5p may target *TNF* and *PTGS2*, while miR-1285-z may target *NFKBIZ*, with *CSF2* predicted as a potential target. This study suggests that chi-miR-148a-5p and miR-1285-z may participate in the TNF and IL-17 signaling pathways by regulating their predicted target genes. Moreover, the TNF and IL-17 signaling pathways are among the most highly enriched immune-related pathways involved in the innate immune response of caprine bronchial epithelial cells. This study provides insights into the molecular mechanisms underlying immune responses in goats infected with *K. pneumoniae*.

## Simple Summary

Clinically, infection with *Klebsiella pneumoniae* in goats can lead to respiratory diseases, including pneumonia and bronchopneumonia. To explore the immune response and elucidate its potential molecular regulatory network, caprine bronchial epithelial cells were infected with *Klebsiella pneumoniae* in this study. We found that *PTGS2*, which is involved in both the TNF and IL-17 signaling pathways, may be regulated by chi-miR-148a-5p. Understanding the innate immune response to *Klebsiella pneumoniae* in caprine bronchial epithelial cells provides a theoretical reference for research on prevention and control of respiratory diseases in goats.

## 1. Introduction

*Klebsiella pneumoniae* (*K. pneumoniae*) is an opportunistic Gram-negative pathogen belonging to the family *Enterobacteriaceae* and represents a major cause of community- and healthcare-, and livestock-associated infections. This bacterium can interact with gut microbiota and colonize the gastrointestinal mucosa of humans and animals [1]. When host immune function is impaired or intestinal barrier integrity is disrupted, commensal *K. pneumoniae* colonizing the gut can translocate into normally sterile internal tissues or be transmitted to new individuals through the fecal–oral pathway [2]. *K. pneumoniae* is widely distributed in natural environments and healthcare-associated settings, including contaminated medical equipment. It is capable of triggering a spectrum of infectious disorders, ranging from urinary tract infections and cystitis to pneumonia, pyogenic liver abscess and endogenous endophthalmitis [3,4,5]. *K. pneumoniae* is one of the ESKAPE pathogens and represents a major global health challenge due to its extensive antimicrobial resistance and rapid acquisition of multidrug-resistant phenotypes [6]. Increasing emergence of hypervirulent and carbapenem-resistant *K. pneumoniae* strains has raised serious concerns due to their enhanced virulence, antimicrobial resistance, and transmission potential [7]. Increasing evidence reports indicate that goats are also hosts of *K. pneumoniae* and can carry highly virulent and drug-resistant strains [8,9,10]. Goats may develop pneumonia, sepsis, and other complications following *K. pneumoniae* infection, and severe cases may result in death [11,12]. This not only causes substantial economic losses to farms but also threatens animal health. Therefore, understanding the pathogenic mechanisms and host responses to *K. pneumoniae* infection, particularly in livestock species, is critical for controlling disease transmission and improving animal health.

The respiratory epithelium represents the primary interface between *K. pneumoniae* and the host, serving not only as a dynamic physical barrier but also as an active immune regulatory interface. Airway epithelial cells participate in host defense and the pathogenesis of inflammatory airway diseases by recognizing pathogens, expressing adhesion molecules, and secreting cytokines and chemokines, thereby regulating the recruitment and activation of inflammatory cells [13]. During *K. pneumoniae* infection, outer membrane vesicles released by the bacterium may be involved in the NF-κB signaling pathway in bronchial epithelial cells, leading to increased expression of pro-inflammatory mediators, including IL-8, IL-6, IL-1β, and TNF-α [14]. Although airway epithelial responses to *K. pneumoniae* have been investigated in humans, the molecular mechanisms underlying the responses of Caprine bronchial epithelial cells (CBECs) to *K. pneumoniae* infection remain largely unknown.

CBECs constitute the first line of defense against respiratory pathogens in goats and have been widely used as an important model for investigating host–pathogen interactions in the respiratory tract. Previous studies have demonstrated that CBECs initiate immune responses through diverse defense mechanisms and undergo significant molecular and cellular alterations following infection with various pathogens, including goat parainfluenza virus type 3 and *Pasteurella multocida* serotypes A and D [15,16,17]. For example, CBECs may regulate membrane cholesterol homeostasis to influence goat parainfluenza virus type 3 infection, while transcriptomic analyses have revealed that different *P. multocida* serotypes activate distinct immune-related signaling pathways, including Toll-like receptor, phagosome, and B-cell receptor pathways [15,16]. In addition, pathogen infection induces morphological alterations in CBECs, including widened intercellular spaces, disruption of cobblestone-like morphology, cell rounding, and detachment, indicating impaired epithelial barrier integrity [16,17].

Research on the immune mechanisms underlying *K. pneumoniae* infection in goats remains relatively limited. Integrated miRNA–mRNA sequencing analysis provides an effective approach for identifying regulatory networks, functional pathways, and key molecular targets involved in host immune responses.

This strategy has been well established in CBECs infected with *P. multocida*—another major respiratory bacterial pathogen threatening goat production. In the in vitro CBEC infection model, parallel mRNA and small RNA sequencing has identified distinct immune regulatory profiles for serotype A and D strains of *P. multocida*. Key differentially expressed immune-related mRNAs include *YPEL2* and *YPEL4*, involved in the Toll-like receptor signaling pathway, *GNB3*, *ADCY7*, and *SLC38A4*, associated with the GABAergic synapse pathway (serotype A-specific), and *LOC102184859* enriched in both the phagosome and B cell receptor signaling pathways (serotype D-specific). The shared transcription regulator KLF4, which modulates interleukin-8 and chemokine production, is also dysregulated in both infection groups. Correspondingly, a panel of regulatory miRNAs has been characterized and predicted to target these immune mRNAs to form post-transcriptional regulatory circuits. Notably, bta-miR-138 is predicted to simultaneously target *ADCY7*, *SLC38A4*, and *YPEL4* to participate in Toll-like receptor-mediated immune regulation, while chi-miR-542-3p targets *LOC102184859* to modulate the serotype D-specific immune response [16].

Current research on *K. pneumoniae* infection in goats has mainly focused on pathogen isolation, identification and antimicrobial resistance profiling. However, the early molecular events and post-transcriptional regulatory mechanisms underlying CBECs’ responses to *K. pneumoniae* infection remain largely unknown. Thus, in this study, a goat clinical isolate of *K. pneumoniae* was used to infect CBECs. The mRNA and miRNA profiles of the control and infection groups at 4 h post-exposure were characterized by mRNA sequencing and miRNA sequencing, followed by integrated bioinformatics analysis. This study aimed to characterize the transcriptomic and post-transcriptional regulatory landscape of CBECs following *K. pneumoniae* infection and provide new insights into host–pathogen interactions.

## 2. Materials and Methods

### 2.1. Cells and Bacteria

CBECs were purchased from Hefei Wanwu Biotechnology Co., Ltd. (Hefei, China, Catalog No.: Delf-27085). Cells at passages 5–6 were used for all experiments in this study and cultured in a caprine bronchial epithelial cell-specific medium supplemented with 6% (*v*/*v*) matching medium components (also from the same company). Cells were maintained at 37 °C in a 5% CO_2_ atmosphere. Caprine-derived *K. pneumoniae* (strain KPHN001) was isolated from lung tissue of a Hainan black goat with clinical pneumonia in our laboratory and confirmed by 16S rRNA sequencing and species-specific PCR [18]. Bacteria were revived and propagated using Tryptic Soy Agar (TSA) and Tryptic Soy Broth (TSB).

### 2.2. Bacterial Infection

CBECs were seeded into 100 mm culture dishes 12 h before infection (*N* = 6, 1.5 × 10^6^ cells per dish) and divided into an experimental group (*N* = 3) and a control group (*N* = 3). An MOI of 100 was selected for bacterial challenge based on preliminary laboratory tests and published protocols for Gram-negative bacterial infection of CBECs [16,17]. This dose was chosen to induce robust early immune transcriptional responses within 4 h of exposure, while avoiding severe cytotoxic injury to the cells. Cells in the experimental group were treated with 100 μL of log-phase KPHN001 at the indicated MOI, while those in the control group received an equal volume of cell-specific medium. After gentle mixing, the cells were incubated for 4 h. Cell morphology was observed under an inverted microscope to assess cell status at the end of infection. The infected cells largely maintained intact epithelial morphology, with no obvious massive cell death or detachment observed. Cell culture supernatants were collected for subsequent PCR detection of bacterial DNA to verify consistent bacterial exposure across replicates. The cells were rinsed three times with PBS, lysed in 6 mL Trizol (Tiangen Biochemical Technology Co., Ltd., Beijing, China), transferred into 15 mL centrifuge tubes, and shipped on dry ice to Guangzhou Genedenovo Biotechnology Co., Ltd., Guangzhou, China, where sequencing was performed using the Illumina NovaSeq X Plus (San Diego, CA, USA) sequencing platform.

### 2.3. RNA Sequencing

Total RNA was extracted using the standard method with Trizol reagent. The integrity of the RNA was confirmed by agarose gel electrophoresis, and the RNA concentration was determined. mRNA was then enriched using oligo(dT) capture beads, purified, and thermally fragmented. Using fragmented mRNA as the template, first-strand cDNA was synthesized by reverse transcription, followed by second-strand cDNA synthesis, concurrent end repair and A-tailing. Following adapter ligation, target fragments were purified and size-selected using Hieff NGS^®^ DNA Selection Beads, and sequencing libraries were constructed by PCR amplification. Libraries passing quality control were sequenced on the Illumina NovaSeq X Plus platform (San Diego, CA, USA). For small RNA sequencing, small RNAs of 18–30 nt were recovered by PAGE electrophoresis, and 3′ and 5′ adapters were ligated sequentially. Reverse transcription was performed followed by PCR amplification. Approximately 140 bp fragments were gel-extracted, eluted in EB buffer, and used for final library construction. Library quality was validated using an Agilent 2100 Bioanalyzer (Agilent Technologies, Santa Clara, CA, USA) and an ABI StepOnePlus Real-Time Quantitative PCR System (Thermo Fisher Scientific, Waltham, MA, USA), followed by sequencing on the Illumina NovaSeq X Plus (San Diego, CA, USA) platform.

### 2.4. mRNA Sequencing Analysis

Raw reads underwent quality control and adapter trimming using fastp to generate clean data. Subsequently, the base composition and quality distribution of the clean data were evaluated. Clean reads were mapped to the NCBI goat reference genome (GCA_040806595.1) using HISAT2 v2.1.0. Transcripts were reconstructed with StringTie v1.3.4, and gene expression levels were quantified using RSEM 1.2.19. Sample reproducibility was assessed by principal component analysis (PCA) and Pearson correlation coefficient analysis based on expression profiles. DEGs were identified using DESeq2 v1.20.0 with read counts as input, with the screening criteria set at *p* < 0.05 and |log_2_ fold change| ≥ 1.5. GO functional annotation and KEGG pathway enrichment analysis were performed on DEGs, and the top 20 significantly enriched terms were identified using the hypergeometric test. GO terms were classified into three categories: biological process (BP), molecular function (MF), and cellular component (CC).

### 2.5. miRNA Sequencing Analysis

Sequenced small RNA reads were referred to as tags. Raw reads were filtered to generate high-quality clean tags, which were then annotated against rRNA, scRNA, snoRNA, snRNA and tRNA sequences from the GenBank and Rfam databases to remove these non-target RNAs. Remaining tags were mapped to the NCBI goat reference genome (GCA_040806595.1), and mRNA degradation fragments were removed based on exon and intron annotations. Species-specific known miRNAs and cross-species conserved homologous miRNAs were both identified by alignment against the miRBase database using bowtie (version 1.1.2). The parameters -v 0 --best --strata -a were adopted for species-specific known miRNAs, whereas -v 2 --best --strata -a were used for cross-species conserved homologous miRNAs. Novel miRNAs were predicted by miRDeep2 based on their characteristic hairpin structures in the reference genome. The expression levels of miRNAs in each sample were quantified using TPM (tags per million). miRNAs with TPM < 1 were filtered out, and sample reproducibility was assessed by principal component analysis (PCA). Differentially expressed miRNAs (DE miRNAs) were identified using the edgeR 3.12.1 software, with a screening threshold set at *p* < 0.05 and |log_2_ fold change| ≥ 1.5. Integrated miRNA-mRNA analysis was performed, and target genes of DEmiRNAs were predicted using miRanda v3.3a and TargetScan v7.0. miRNA-target gene regulatory relationships were established, and GO functional and KEGG pathway enrichment analyses were conducted on predicted target genes of DEmiRNAs. GO terms were grouped into biological process (BP), molecular function (MF), and cellular component (CC). Finally, the miRNA-target gene regulatory network was visualized using Cytoscape v3.10.4 software.

### 2.6. RT-qPCR Validation

DEGs and DEmiRNAs identified from sequencing were subjected to RT-qPCR validation. DEGs-specific primers were designed using the NCBI Primer-BLAST tool (https://www.ncbi.nlm.nih.gov/tools/primer-blast, accessed on 2 December 2025) (Table S1), whereas the primers for DEmiRNAs were designed using Primer Premier 5.0 (Table S2). Per the supplier’s instructions, total RNA was reverse transcribed into cDNA using the FastKing One-step gDNA Removal and cDNA First-strand Synthesis Premix Reagent (Tiangen Biotech (Beijing) Co., Ltd., Beijing, China) (Table S3). RT-qPCR was performed using the SYBR Green Pro Taq HS Premix qPCR Kit (with tracking dye and ROX reference dye) (Aikerui Biotechnology Co., Ltd., Changsha, Hunan, China) on the QuantStudio™ 5 Real-Time PCR System (Applied Biosystems, Waltham, MA, USA). Detailed information regarding the RT-qPCR reaction system and thermal cycling conditions is listed in Tables S4 and S5. DEGs were identified using bioinformatic analyses. The expression levels of these DEGs were visualized using BioLadder (https://www.bioladder.cn), and the protein–protein interaction (PPI) network of the DEGs was constructed using the STRING database (https://cn.string-db.org) and Cytoscape software. The reverse transcription system for miRNAs was prepared following the formulations listed in Table S6. All RT-specific primers applied in this experiment were synthesized by Shanghai BBI Biotechnology Co., Ltd. (Shanghai, China), as detailed in Table S7. The reaction was incubated sequentially at 16 °C for 30 min, 42 °C for 40 min, and 85 °C for 5 min to generate cDNA, which was subsequently used as the template for RT-qPCR detection of DEmiRNAs. DEmiRNA RT-qPCR was conducted using the SYBR^®^ Green qPCR Master Mix (Rox Plus; Arraystar, Rockville, MD, USA) on the ViiA 7 Real-Time PCR System (Applied Biosystems). Detailed information regarding the RT-qPCR reaction system and thermal cycling conditions is listed in Tables S8 and S9. Relative expression levels of DEGs and DEmiRNAs were calculated using the 2^(−ΔΔCt) method, with *GAPDH* and snRNA *U6* serving as internal reference genes, respectively. All RT-qPCR experiments were performed with three independent biological replicates, and each biological replicate included three technical replicates. Results were expressed as mean ± standard error of the mean (SEM), and graphs were generated using GraphPad Prism 8 software. Statistical significance was assessed using unpaired two-tailed *t*-tests.

### 2.7. Validation of miRNA Target Gene Regulation

Based on the quantitative results of miRNAs, chi-miR-148a-5p and miR-1285-z were selected to verify the miRNA-target gene regulation. chi-miR-148a-5p and miR-1285-z mimics and inhibitors were synthesized by Suzhou GenePharma Co., Ltd., Suzhou, China. CBECs were seeded into 12-well plates at a density of 1.5 × 10^5^ cells per well, and transfection was performed after 12 h of incubation. Cy3-labeled negative control miRNA mimic, along with chi-miR-148a-5p mimic and miR-1285-z mimic, was centrifuged at 4000 rpm for 1 min, then diluted to 20 μM with DEPC-treated water and mixed thoroughly. For transfection, RNAiMAX reagent was gently mixed. Solution A and Solution B were prepared separately in 1.5 mL EP tubes, then combined to form a 200 μL transfection complex, which was incubated at room temperature for 18 min (Table S10). One hour prior to transfection, the medium in the 12-well plates was replaced with 800 μL of serum-reduced medium. After 1 h, 200 μL of the transfection mixture was added to each well, and the cells were cultured in an incubator. Six hours later, the medium was exchanged for complete serum-containing media. Cells transfected with the Cy3-labeled negative control were visualized under an inverted fluorescence microscope. The remaining plates were returned to the incubator, and samples were collected 48 h post-transfection. miRNA inhibitor transfection was performed using the same protocol. Images including bright-field (Light), FAM fluorescence, and Cy3 fluorescence single-channel micrographs were acquired via fluorescence microscopy and subsequently imported into ImageJ 1.53t software. Bright-field and fluorescent signals were overlaid and fused (Merge) to generate composite images, and standardized scale bars were applied to all panels within ImageJ. Finally, the expression levels of potential miRNA target genes were validated by RT-qPCR. Each RT-qPCR experiment was run in three technical replicates. Results were expressed as mean ± standard error of the mean (SEM), and graphs were generated using GraphPad Prism 8 software. Statistical significance was assessed using unpaired two-tailed *t*-tests.

## 3. Results

### 3.1. CBECs Infected with K. pneumoniae

**Figure 1:**
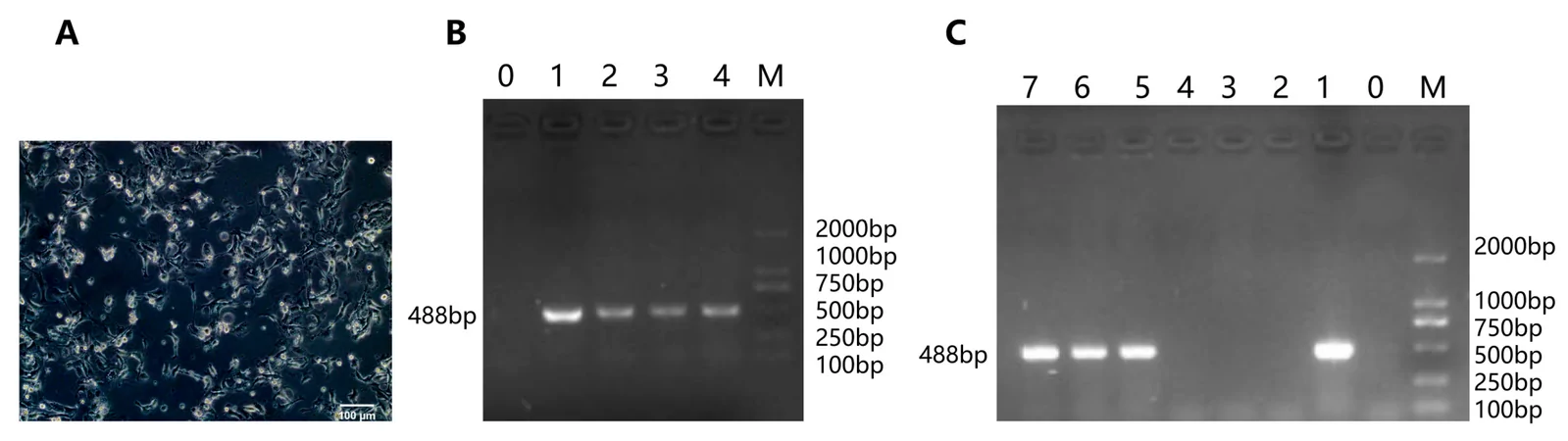
CBECs infected with *K. pneumoniae.* (**A**) Morphological characteristics of in vitro-cultured CBECs (scale bar = 100 μm). (**B**) Specific PCR identification of strain KPHN001. Lane 0: Negative control, ddH_2_O (sterile water) instead of bacterial suspension was added to the PCR system; Lane 1: Positive control, the genomic DNA of *K. pneumoniae* served as the PCR template; Lanes 2–4: KPHN001; Lane M: D2000 marker. (**C**) PCR detection of *K. pneumoniae* DNA in cell culture supernatants. Lane M: D2000 DNA marker; Lanes 1–3: three replicate samples from the experimental group; Lanes 4–6: three replicate samples from the control group; Lane 7: positive control (*K. pneumoniae* genomic DNA as template); Lane 8: negative control (sterile ddH_2_O as template).

CBECs displayed a polygonal and cobblestone morphology (Figure 1A). Before infection, bacterial suspensions were identified using *K. pneumoniae Khe* gene-specific primers [18]. Three randomly picked colonies yielded the expected amplicon (Figure 1B), verifying the isolate as *K. pneumoniae*. PCR was performed with supernatants collected 4 h post-incubation from control and experimental groups. The target band was detected in the infection group, confirming the presence of bacterial DNA in the supernatant and consistent bacterial exposure across experimental replicates (Figure 1C).

### 3.2. Sequencing Evaluation and Differential Expression Analysis

The control group was designated as C, and the infection group as E. After quality filtering, an average of 44,759,864 clean reads were generated per sample, with Q30 > 93.75% and an average GC content of 49.21%. Mapped reads accounted for 92.02–92.34% of all clean reads against the reference genome. These datasets were valid for subsequent transcript assembly and gene expression quantification (Table 1).

**Figure 2:**
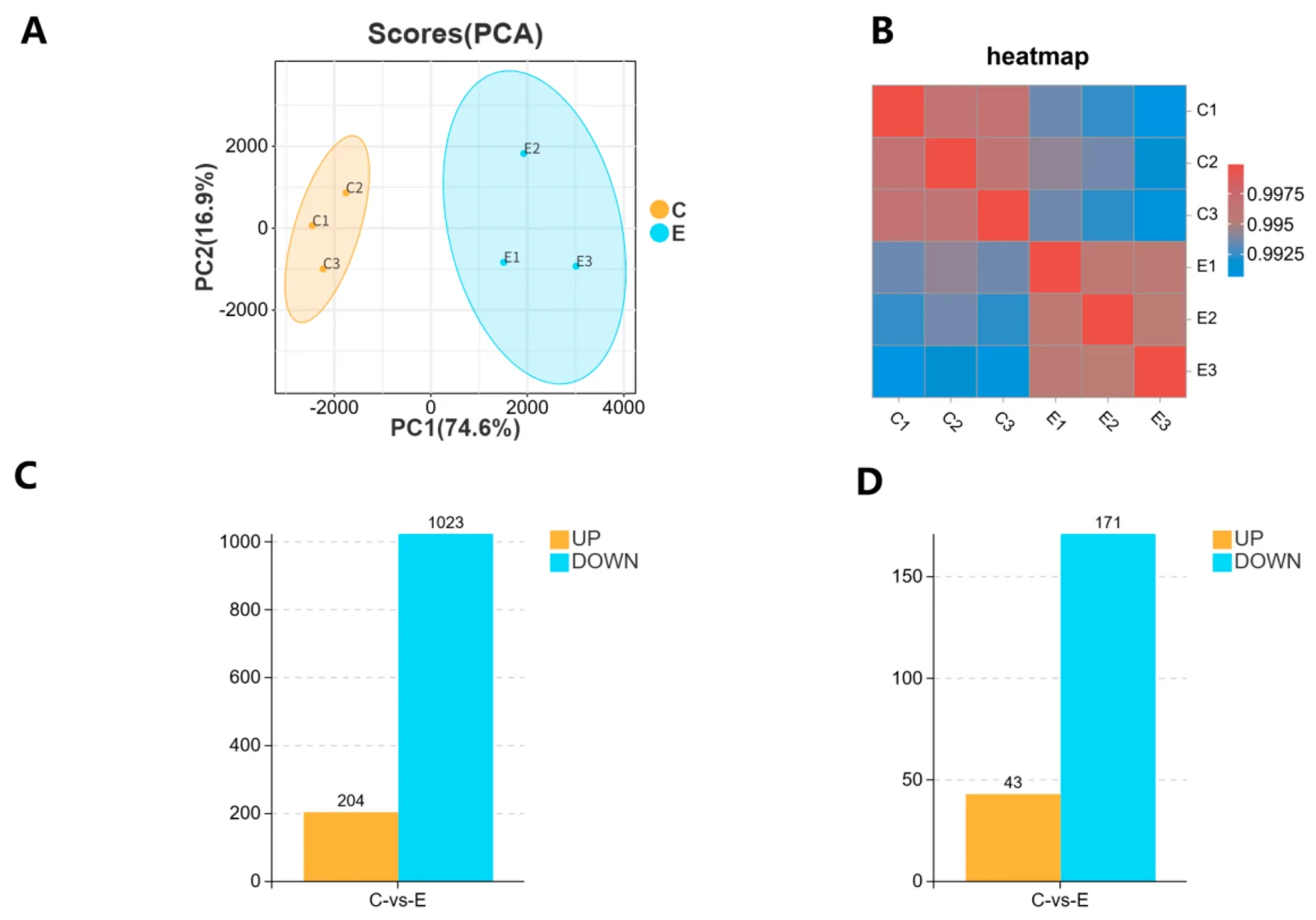
Correlation and differential expression analysis of sequenced samples. (**A**) Principal component analysis (PCA) of sequenced samples. The samples in group C and group E are represented by orange and blue dots, respectively. (**B**) Correlation heat map of sequenced samples. The color bar from red to blue indicates that the correlation between samples is from strong to weak. (**C**) Histogram of DEGs between group C and group E. (**D**) Histogram of DEmiRNAs between group C and group E.

Based on the principal component analysis (Figure 2A), the control and experimental groups were significantly separated on PC1, which explained 74.6% of the variance, indicating substantial differences between the two groups. Sample correlation analysis (Figure 2B) showed high intra-group correlations close to 1 and low inter-group correlations, confirming good within-group consistency and reliable experimental reproducibility. We identified DEGs (Figure 2C), including 204 up-regulated and 1023 down-regulated genes, totaling 1227 DEGs. We also identified DE miRNAs (Figure 2D), including 43 up-regulated and 171 down-regulated miRNAs, for a total of 214 DE miRNAs.

### 3.3. GO and KEGG Enrichment Analysis

**Figure 3:**
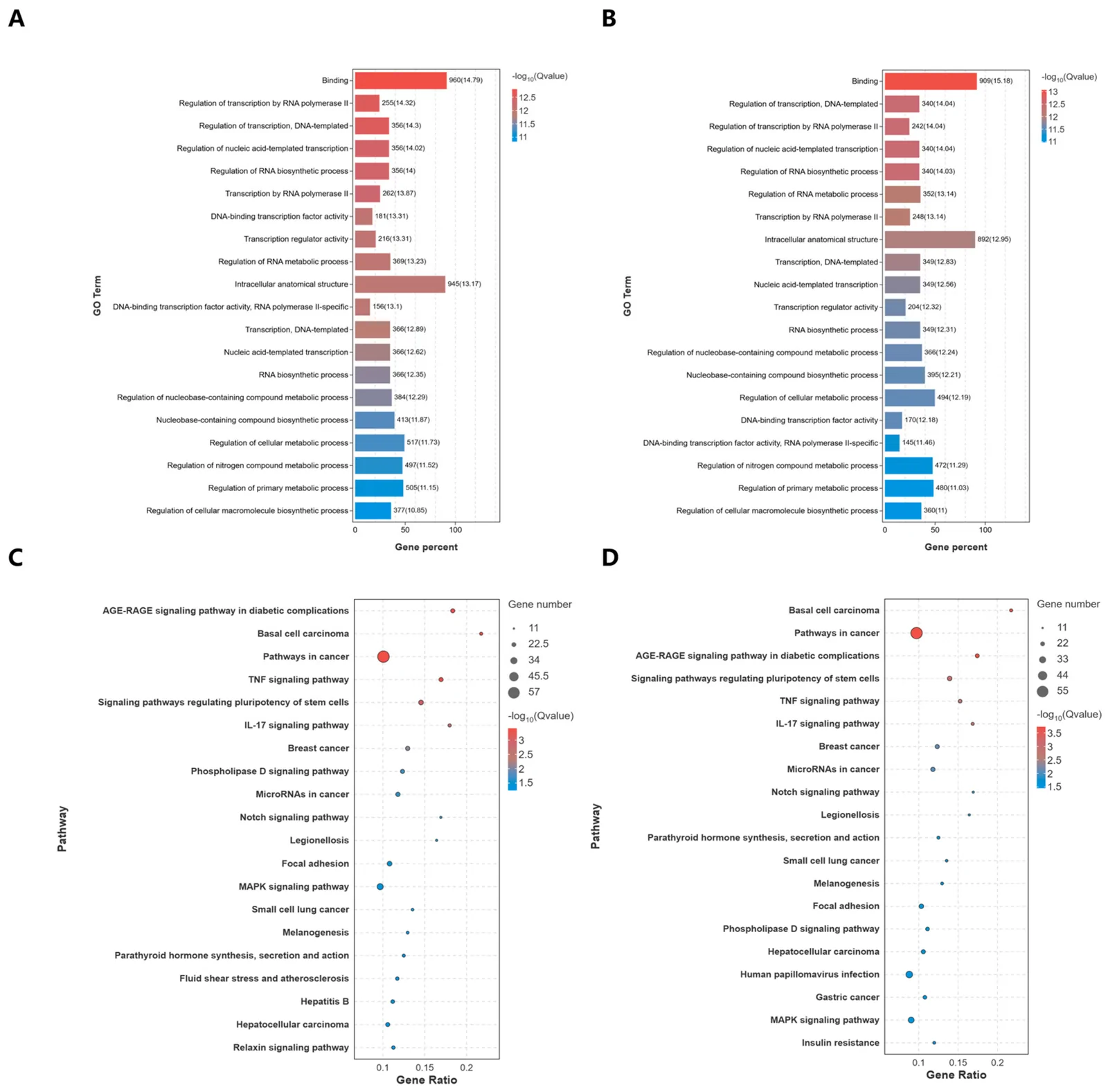
GO and KEGG enrichment analyses of DEGs and predicted target genes of DEmiRNAs (**A**) GO enrichment scatter plot for DEGs (group C vs. group E), categorized into biological process (BP), molecular function (MF), and cellular component (CC). (**B**) GO enrichment scatter plot for predicted target genes of DEmiRNAs (group C vs. group E), categorized into BP, MF, and CC. (**C**) KEGG enrichment histogram of DEGs between Group C and Group E. (**D**) KEGG enrichment histogram of target genes of DEmiRNAs between Group C and E. The top 20 GO entries with significant enrichment were displayed (*p* < 0.05). The top 20 significantly enriched KEGG pathways are shown (*p* < 0.05). Gene percent and Gene ratio represent the proportion of enriched DEGs or target genes of DEmiRNAs in corresponding pathways relative to the total number of background genes.

GO enrichment analysis of DEGs (Figure 3A) and target genes (Figure 3B) showed highly consistent patterns, with significant enrichment mainly in key biological processes including binding, transcriptional regulation, RNA biosynthesis, and cellular metabolic regulation. These findings indicate that miRNA-mediated regulation specifically targets the important candidate biological processes underlying mRNA expression changes. 

After *K. pneumoniae* infection of CBECs, among the top 20 enriched pathways of DEGs and DEmiRNA target genes (Figure 3C,D), tumor, metabolism, signal transduction and immune pathways were enriched. The immune-related TNF and IL-17 signaling pathways were markedly enriched, suggesting that they may represent important candidate immune pathways involved in the response to *K. pneumoniae* infection. Further functional experiments are required to confirm their direct activation.

### 3.4. RT-qPCR Verification of DEGs

**Figure 4:**
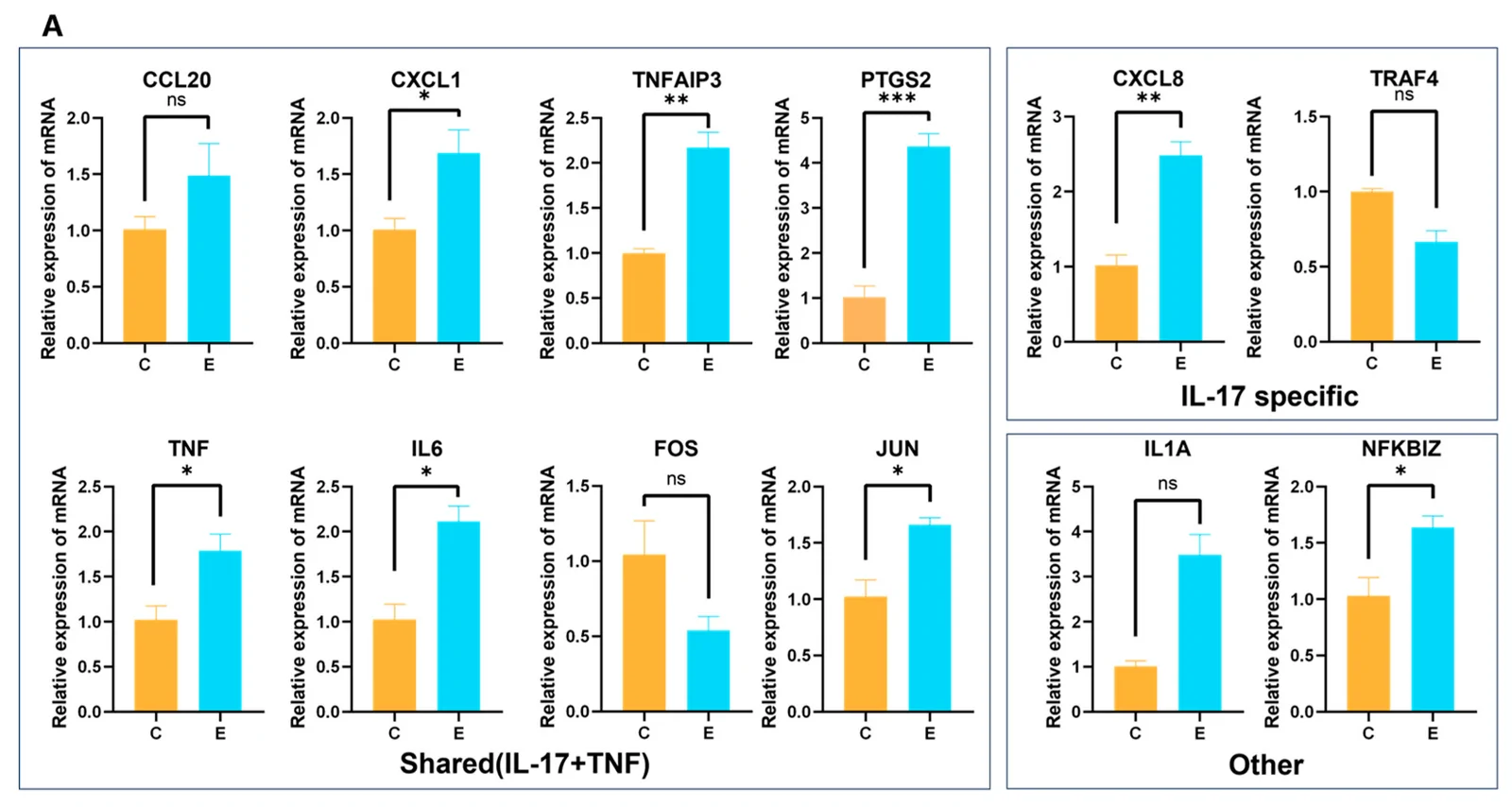

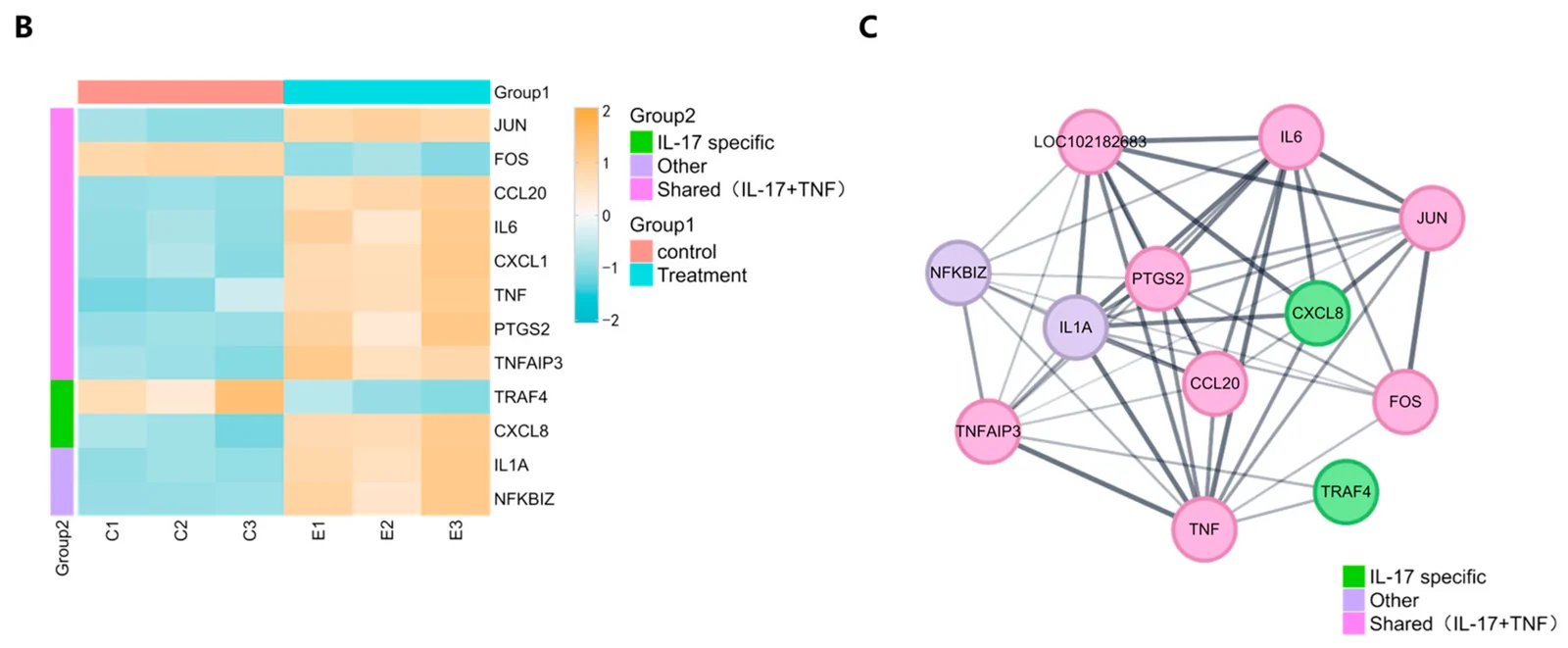
RT-qPCR validation and bioinformatics analysis of DEGs involved in IL-17 and TNF signaling pathways. (**A**) RT-qPCR validation of selected DEGs. (**B**) Expression heatmap of DEGs. (**C**) Protein–protein interaction (PPI) network of DEGs. Group C and E represent control and experimental samples, respectively. Data are presented as mean ± standard error (SEM). Unpaired two-tailed *t*-tests were used for comparisons. * indicates *p* < 0.05, ** indicates *p* < 0.01, *** indicates *p* < 0.001, and ns represents no significant difference.

To verify the reliability of the mRNA-seq DEG expression trends, 10 immune-related genes (*CCL20*, *CXCL1*, *TRAF4*, *FOS*, *IL6*, *PTGS2*, *TNF*, *CXCL8*, *TNFAIP3*, *JUN*) from the TNF and IL-17 signaling pathways were selected for RT-qPCR verification. The important candidate genes in these two pathways may be candidate targets for further research. RT-qPCR results showed that the expression levels of 8 genes increased and those of 2 genes decreased, which was consistent with the expression trends observed in the RNA-seq data. Subsequently, two additional DEGs, *IL1A* and *NFKBIZ*, were randomly selected to conduct RT-qPCR verification to further assess the reliability of transcriptome sequencing outcomes. RT-qPCR confirmed that both genes were up-regulated. These results indicate that the quantitative RT-qPCR results (Figure 4A) are consistent with the expression trends observed in the sequencing data (Figure 4B). Additionally, the constructed protein–protein interaction (PPI) network revealed complex functional interrelationships among these DEGs (Figure 4C). Notably, important candidate hub genes including *TNF*, *IL1A*, *FOS*, and JUN directly interact with numerous other genes in the network.

### 3.5. Construction of the miRNA–DEG Regulatory Network

**Figure 5:**
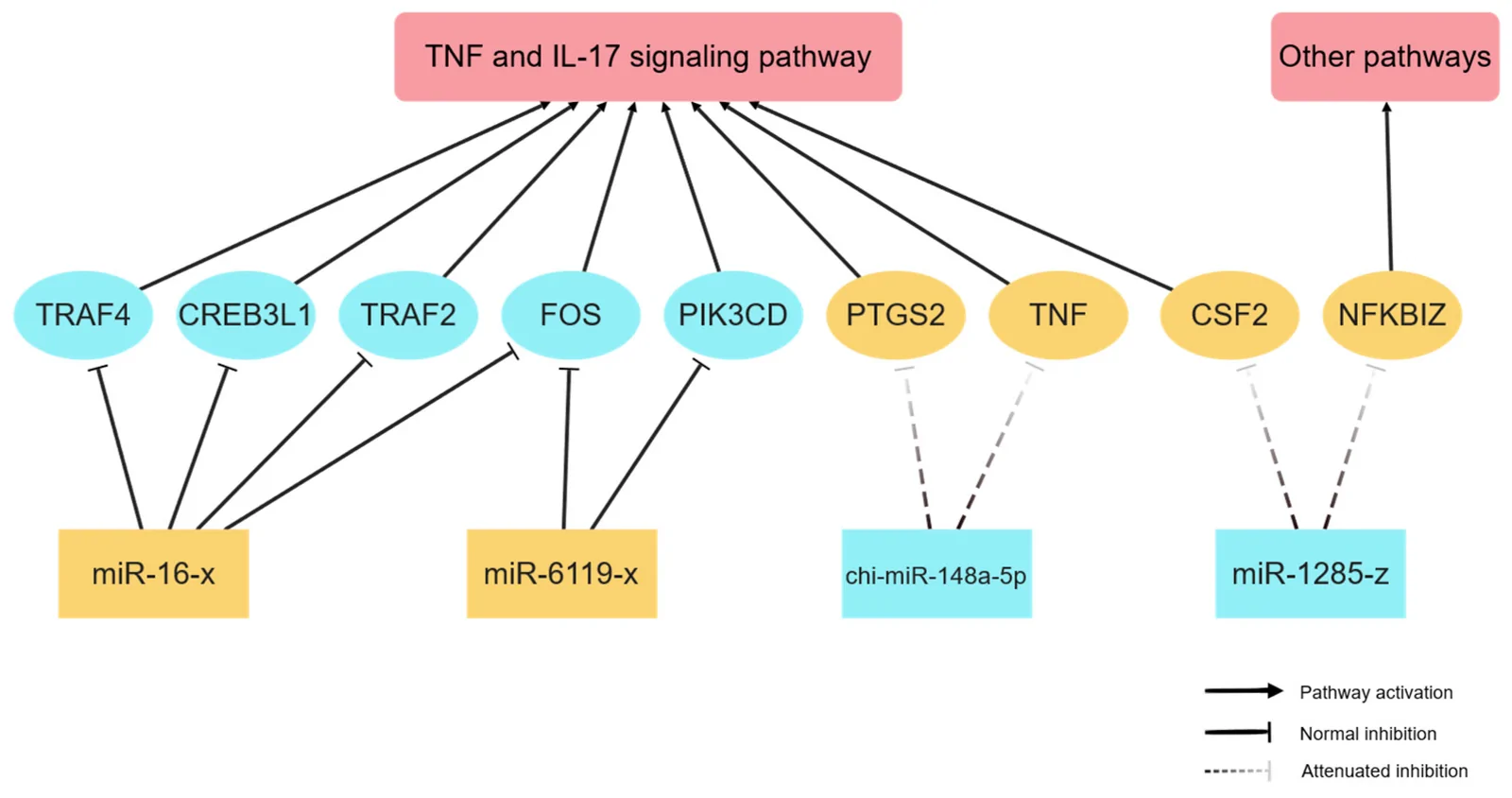
miRNA-target gene interaction network. Blue nodes indicate downregulated transcripts, and yellow nodes indicate upregulated transcripts. Solid arrows mark gene participation in corresponding pathways, solid vertical bars show basal miRNA-mediated inhibition, while dashed lines illustrate reduced inhibitory capacity due to miRNA downregulation.

Integrated analysis of transcriptome and miRNA sequencing data suggested that four differentially expressed miRNAs (miR-16-x, miR-6119-x, chi-miR-148a-5p, and miR-1285-z) may be involved in the regulation of the TNF and IL-17 signaling pathways (Figure 5). Bioinformatic prediction suggested that upregulated miR-16-x and miR-6119-x may target a panel of genes including *TRAF2*, *CREB3L1*, *TRAF4*, *FOS*, and *PIK3CD*. However, this predicted inhibitory relationship has not been experimentally validated in this study. In contrast, chi-miR-148a-5p was predicted to target *PTGS2* and *TNF*, while miR-1285-z was predicted to regulate *CSF2* and *NFKBIZ*. It should be noted that direct physical binding between these miRNAs and their putative target mRNAs has not been verified in the current study. The attenuation of miRNA-mediated inhibitory effects induced by their downregulation may contribute to the significant upregulation of *PTGS2*, *TNF*, *CSF2* and *NFKBIZ*. Notably, all these target genes are enriched in the two aforementioned pathways, except for *NFKBIZ*, which has documented functional roles in other biological processes.

**Figure 6:**
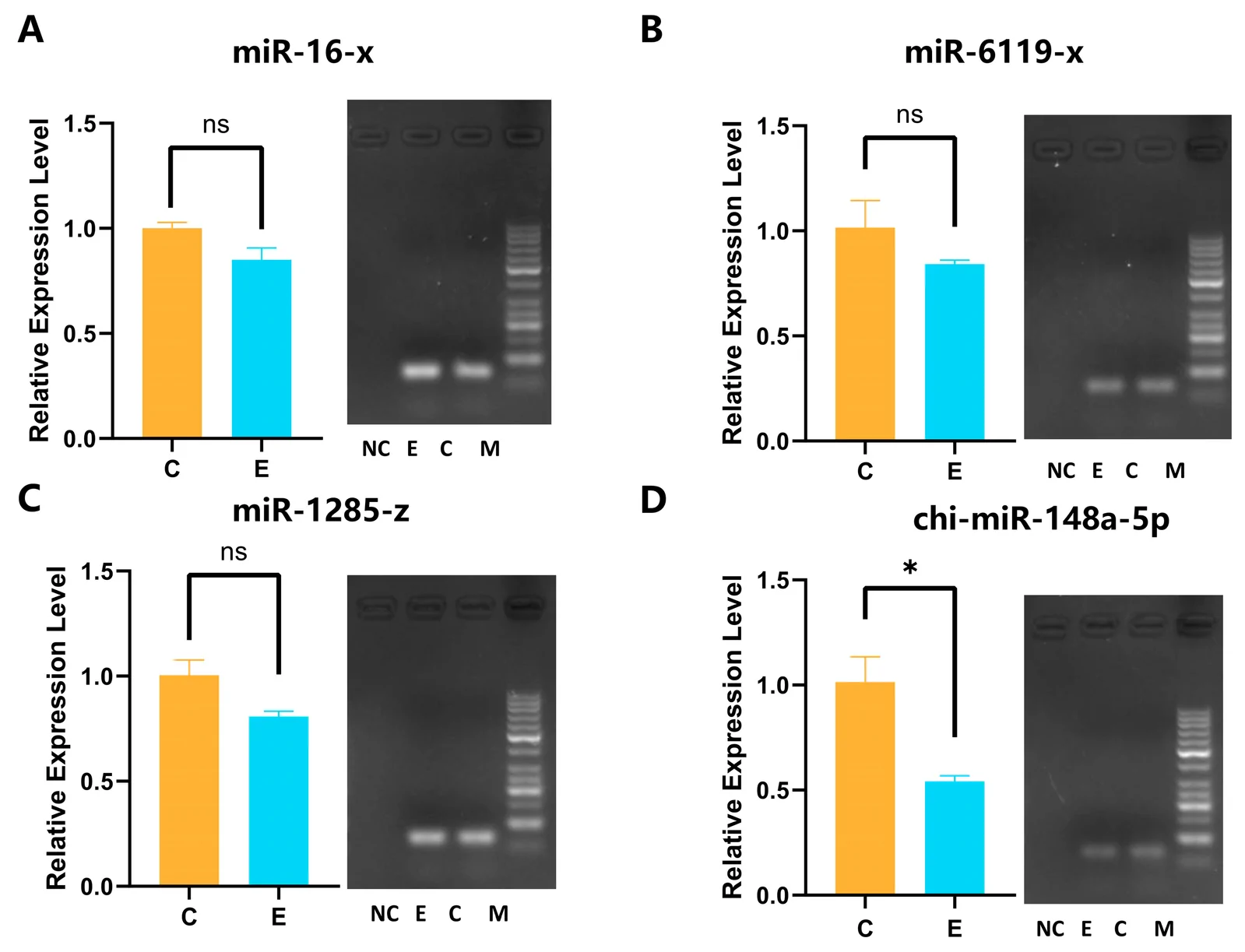
RT-qPCR validation of four differentially expressed miRNAs. (**A**) miR-16-x; (**B**) miR-6119-x; (**C**) miR-1285-z; (**D**) chi-miR-148a-5p. Left panels: relative expression of miRNAs after K. pneumoniae stimulation. Right panels: agarose gel electrophoresis results corresponding to RT-qPCR products. Lane C: group C; Lane E: group E; Lane NC: negative control; Lane M: DNA Marker (50–1031 bp). Data are presented as mean ± standard error (SEM). Unpaired two-tailed *t*-tests were used for comparisons. * indicates *p* < 0.05, and ns represents no significant difference.

The RT-qPCR results of miRNA are shown in Figure 6. The chi-miR-148a-5p and miR-1285-z are consistent with the down-regulation trend of sequencing, while miR-16-x and miR-6119-x RT-qPCR results are down-regulated, which is opposite to the trend of sequencing data.

### 3.6. Validation of miRNA Regulation

**Figure 7:**
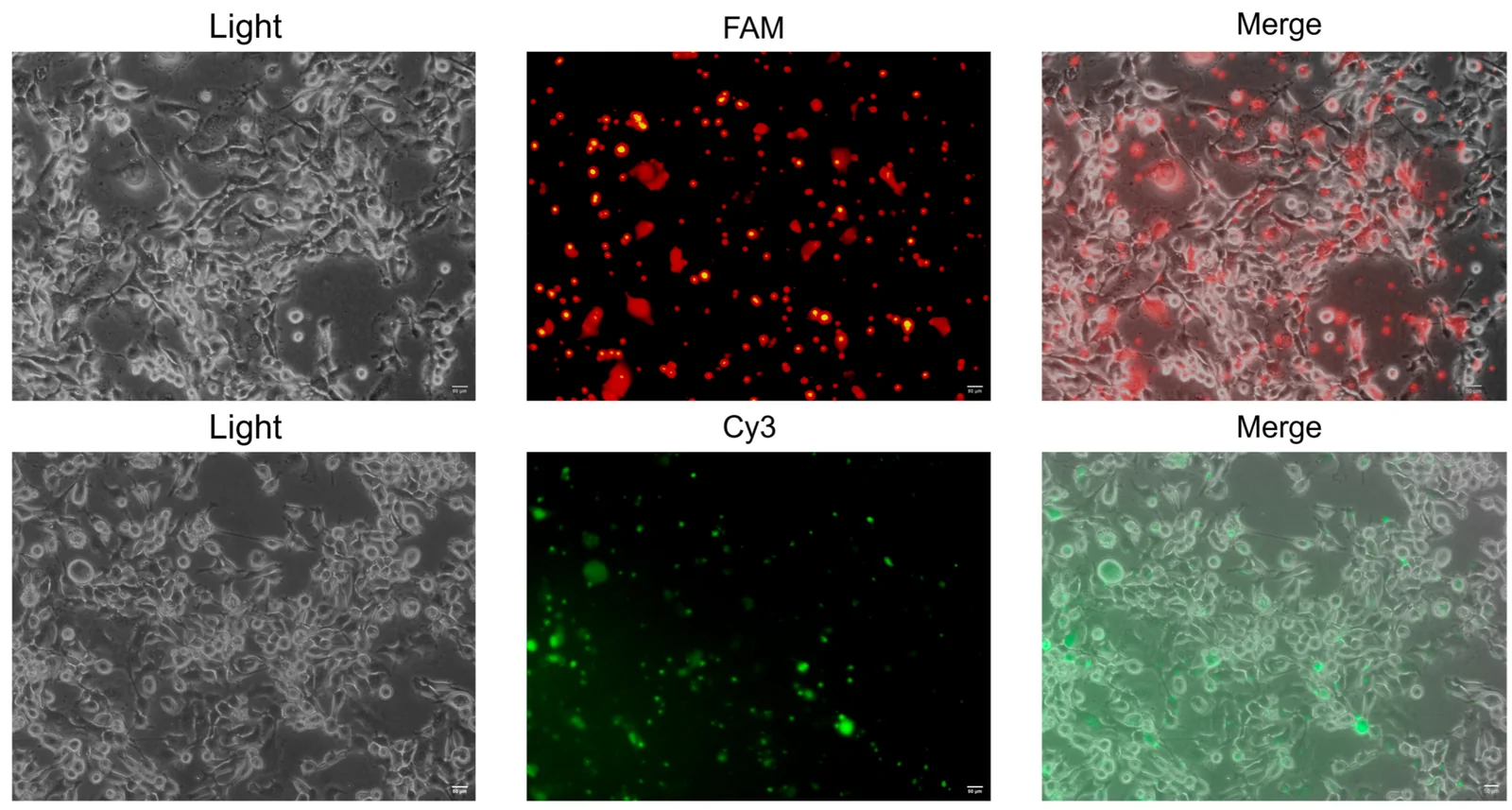
Transfection efficiency of CBECs. Fluorescent expression in CBECs transfected with FAM-labeled negative control miRNA inhibitor and Cy3-labeled negative control miRNA mimic after 6 h, scale bar = 50 μm.

The miRNA-target gene interaction network indicated that chi-miR-148a-5p and miR-1285-z may target genes associated with the TNF and IL-17 signaling pathways. We therefore transfected mimics and inhibitors of these two miRNAs to examine their regulatory effects on the corresponding hub genes. Transfection efficiency was assessed using fluorescence signals from FAM-labeled negative control miRNA inhibitor and Cy3-labeled negative control miRNA mimic. At 6 h after transfection, strong green and red fluorescence was detected in cells under fluorescence microscopy (Figure 7), indicating successful miRNA transfection and supporting the subsequent 48 h functional assays.

**Figure 8:**
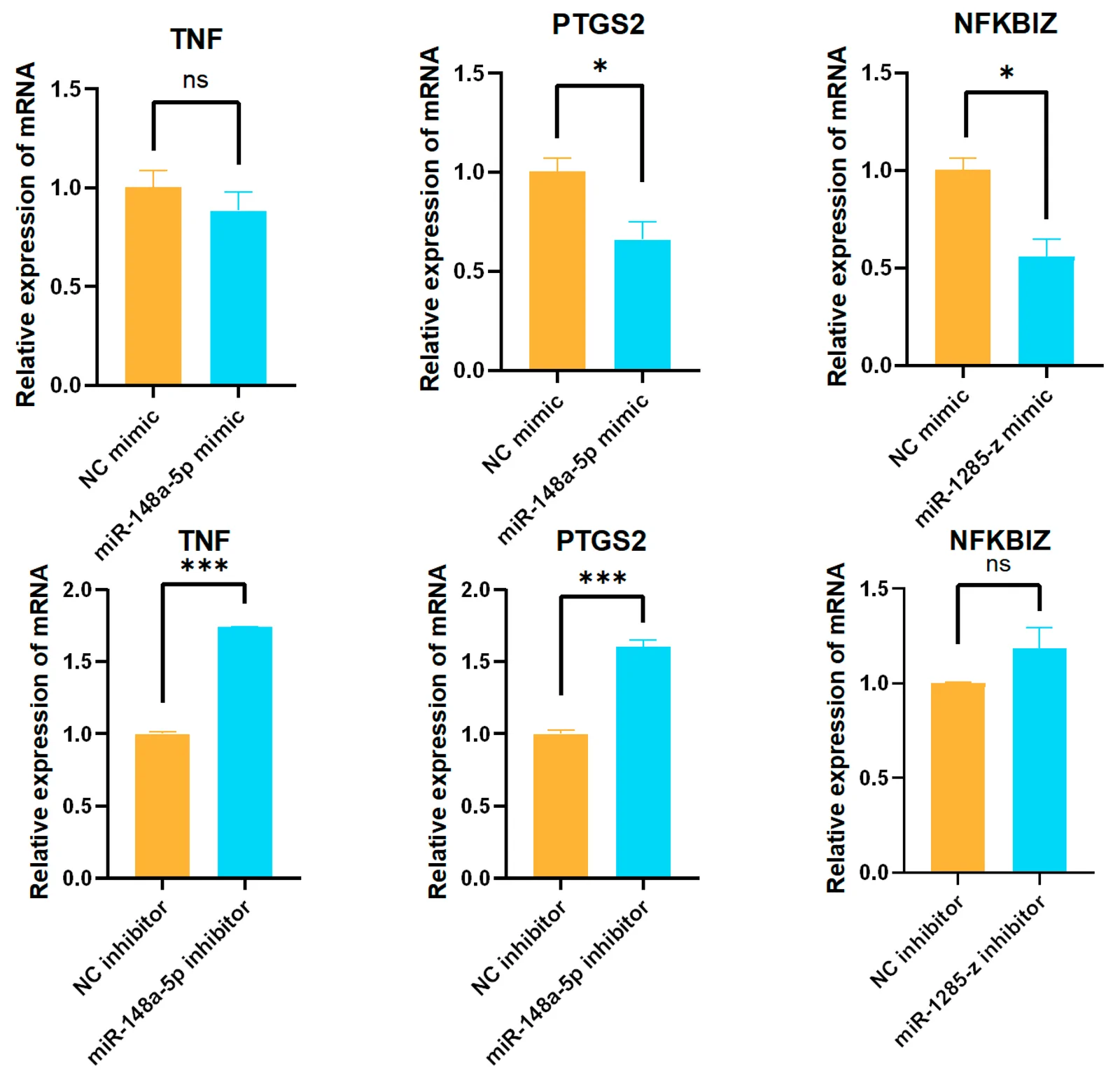
Validation of miRNA-target gene regulation. Data are presented as mean ± standard error (SEM). Unpaired two-tailed *t*-tests were used for comparisons. * indicates *p* < 0.05, *** indicates *p* < 0.001, and ns represents no significant difference.

RT-qPCR analysis of miRNA-target genes revealed that transfection with chi-miR-148a-5p and miR-1285-z mimics reduced the expression of *TNF*, *PTGS2* and *NFKBIZ* (Figure 8). Conversely, treatment with the corresponding inhibitors led to an increase in the expression of these three genes (Figure 8). For the candidate target gene *CSF2*, we did not obtain valid primers suitable for RT-qPCR after repeated design and amplification testing. Consequently, this gene was not included in the verification experiments of this study.

## 4. Discussion

Infection of goats with *K. pneumoniae* causes pneumonia and sepsis, inflicting substantial economic losses on goat farming and posing potential public health risks. As the first line of airway mucosal defense, bronchial epithelial cells orchestrate early innate immune responses against invading pathogens. In this study, we established an in vitro *K. pneumoniae* exposure model using CBECs and integrated mRNA and small RNA sequencing to dissect the post-transcriptional regulatory network underlying host immune responses [16,17,19,20]. The MOI of 100 was selected based on previous laboratory studies and published bacterial challenge protocols for respiratory epithelial cells. 

MiRNAs function in post-transcriptional repression by pairing with target mRNAs through the RNA-induced silencing complex (RISC), thereby widely regulating gene expression [21,22]. Bioinformatic prediction suggested that chi-miR-148a-5p may regulate *PTGS2* and *TNF* expression, whereas miR-1285-z may regulate *NFKBIZ* and *CSF2* expression. The miRNA-seq results were consistent with those obtained by RT-qPCR. *PTGS2*, as a stress-induced inflammatory regulatory enzyme, exhibits low expression under physiological conditions and can be directly induced and may be involved by pro-inflammatory cytokines released and pathogen-associated molecular patterns during infection [23]. As an important candidate component of the respiratory mucosal barrier, the upregulation of *PTGS2* in CBECs essentially represents the host’s rapid inflammatory response to bacterial infection. TNF serves as a key upstream initiator and co-amplifier of infection and inflammation. Upon binding to TNFR1, TNF promotes the formation of membrane-bound complex I, which recruits kinases via K63/M1 ubiquitin chains to activate NF-κB and MAPK pathways. This directly induces transcriptional upregulation of pro-inflammatory factors (e.g., IL-6, CXCL1, IL-8), chemokines, and adhesion molecules, thereby initiating the innate immune response [24]. Consequently, *TNF* also serves as a critical immune mediator in CBECs infected with *K. pneumoniae.* In the early stage of inflammation, NFKBIZ inhibits the excessive release of pro-inflammatory factors including TNF-α, IL-17, and IFN-γ to prevent inflammatory storms. In the late stage of inflammation, NFKBIZ synergistically activates the transcription of downstream target genes such as *IL-6*, *CCL2*, *LCN2*, and *IL-10*, strengthening targeted inflammatory responses and pathogen clearance [25]. After *K. pneumoniae* infected CBECs, *CSF2* expression was significantly upregulated. CSF2 (GM-CSF) serves as an important regulatory factor in inflammatory and infectious conditions, with its expression predominantly localized to inflammatory sites where it exerts pathophysiological effects [26]. The above results indicate that *PTGS2, TNF, NFKBIZ,* and *CSF2*, predicted as potential targets of chi-miR-148a-5p and miR-1285-z, are all involved in the immune response of CBECs upon *K. pneumoniae* exposure.

In the sequencing results, FOS was significantly downregulated and was predicted to be a potential target gene of miR-16-x and miR-6119-x. RT-qPCR validation confirmed that FOS was significantly downregulated, consistent with the sequencing data. However, miR-16-x and miR-6119-x showed a mild downregulation trend by RT-qPCR [27,28] (not statistically significant), which was opposite to the upregulation trend observed in sequencing. These findings indicate that miR-16-x and miR-6119-x are not the primary regulatory molecules responsible for FOS downregulation; FOS downregulation is more likely mediated by other signaling pathways or regulatory factors. The negative regulatory relationship between these miRNAs and FOS is only a bioinformatic prediction and has not been validated at the expression level in this study [29,30,31].

GO enrichment profiling showed that differentially expressed genes were mainly enriched in transcriptional regulation, nucleic acid metabolism, and cellular component-related terms, with binding, transcriptional regulation, and RNA biosynthesis showing high enrichment levels. This suggests that following *K. pneumoniae* infection, cells initiate immune responses at the transcriptional level and adjust metabolic states to cope with the stress microenvironment induced by pathogen invasion. 

Analysis of KEGG signaling pathways revealed a significant increase in the number of DEGs in inflammatory signaling pathways such as TNF and IL-17, suggesting that these may represent important candidate immune pathways involved in the response to *K. pneumoniae* exposure. These pathways may contribute to pro-inflammatory immune responses and local respiratory inflammatory reactions upon bacterial infection. TNF expression was markedly upregulated, and the corresponding signaling pathway was enriched, which is consistent with previous findings in CBECs infected with *P. multocida*, where the *TNF* gene showed specific chromatin accessibility peaks and the TNF signaling pathway was also significantly enriched [17].

Notably, compared with *P. multocida* serotypes A and D, which mainly activate GABAergic synapse and Toll-like receptor pathways or phagosome and B-cell receptor pathways in CBECs [16], *K. pneumoniae* infection in this study showed more prominent enrichment of TNF and IL-17 signaling pathways. In addition, a transcriptomic study of Mannheimia haemolytica-infected goat peripheral blood mononuclear cells also identified TNF and IL-6 as important candidate immune regulatory nodes, with significant enrichment of the JAK-STAT signaling pathway [32]. These cross-pathogen and cross-cell-type comparisons indicate that different bacterial pathogens may trigger distinct dominant immune signatures depending on host cell types, while TNF serves as a shared important candidate pro-inflammatory mediator across multiple respiratory pathogens and immune cellular contexts.

Furthermore, the high expression of IL-6, CXCL1, and CXCL8 in this study may be attributed to downstream effects associated with the IL-17 pathway. Cao et al. demonstrated that IL-17A/F significantly enhances the release of IL-6, CXCL1, and CXCL8 by bronchial epithelial cells [33]. CXCL1 and CXCL8 are critical inflammatory factors produced by bronchial epithelial cells in response to pathogen stimulation [34,35]. These findings suggest that *K. pneumoniae* may induce inflammatory responses in CBECs through the potential activation of the IL-17 signaling pathway. KEGG enrichment analysis showed that *PTGS2*, *TNF*, and *CSF2* were involved in the TNF and IL-17 signaling pathways. Furthermore, *NFKBIZ* serves as an important downstream regulatory gene in the IL-17 signaling pathway, amplifying the biological effects of the IL-17 pathway by upregulating downstream inflammatory mediators and pro-proliferative signals [36,37].

Upon *K. pneumoniae* exposure, CBECs mounted inflammatory and innate immune responses, with marked enrichment of TNF and IL-17 signaling pathways. In line with our findings, previous studies in human bronchial epithelial cells have also reported that the TNF-α/IL-17 signaling axis drives inflammatory responses under stimulatory conditions such as tobacco smoke extract [38]. Similarly, in vivo investigations have confirmed that *K. pneumoniae* infection can induce pulmonary inflammatory injury and disrupt airway epithelial homeostasis in mouse models [20]. These observations collectively indicate that the TNF and IL-17 pathways represent a relatively conserved immune defense module across species in response to respiratory bacterial challenges.

In terms of novelty, this study is the first to construct an integrated miRNA-mRNA regulatory network in *K. pneumoniae*-infected CBECs, and to identify the potential chi-miR-148a-5p-PTGS2 regulatory axis associated with TNF and IL-17 pathways. These findings fill the gap in understanding of miRNA-mediated post-transcriptional regulatory mechanisms during respiratory bacterial infection in ruminants, and provide candidate molecular targets for follow-up functional studies.

This study has several limitations that should be acknowledged. First, all experiments were performed in vitro using cultured CBECs, which cannot fully recapitulate the complexity of in vivo tissue microenvironments and systemic immune regulation. The regulatory mechanisms identified here require further validation in animal infection models. Second, the regulatory relationships between miRNAs and target genes were only verified at the mRNA level; confirmation at the protein level (e.g., Western blot, immunofluorescence) and direct binding evidence (e.g., dual-luciferase reporter assay) are still needed. Third, this study used only one bacterial strain and a single cell type, so the generalizability of the findings remains to be tested with additional strains and experimental systems.

## 5. Conclusions

In conclusion, this study established an in vitro *Klebsiella pneumoniae* infection experiment using CBECs and revealed comprehensive transcriptomic and small RNA responses during bacterial challenge. Integrated analysis identified 1227 differentially expressed mRNAs and 214 differentially expressed miRNAs, with significant enrichment in immune-related processes, particularly the TNF and IL-17 signaling pathways. Further validation suggested that chi-miR-148a-5p and miR-1285-z may contribute to host immune defense against *K. pneumoniae* by regulating key immune-related targets, including *PTGS2*, *TNF*, and *NFKBIZ*. Although this study focused on host–pathogen interactions using a single clinical isolate rather than strain-specific virulence comparison, the findings provide novel insights into the molecular mechanisms of goat respiratory immunity and identify potential regulatory molecules for future functional investigations.

## Figures and Tables

**Table 1:** Sequencing data of each sample.

Sample	Raw Reads	Clean Reads	Mapped Reads (%)	Q30 (%)	GC Content (%)
C1	51,397,702	51,052,810	47,119,257 (92.34%)	93.99%	49.86%
C2	45,548,904	45,265,562	41,683,556 (92.13%)	93.82%	49.76%
C3	45,454,356	45,190,968	41,685,355 (92.30%)	92.88%	49.88%
E1	44,776,272	44,501,892	41,258,843 (92.75%)	93.89%	48.87%
E2	46,603,494	46,311,498	43,026,326 (92.95%)	94.32%	48.77%
E3	36,511,064	36,236,454	33,324,276 (92.02%)	93.75%	48.12%

## Data Availability

The raw data reported in this paper have been deposited in NCBI Sequence Read Archive (https://www.ncbi.nlm.nih.gov/sra, accessed on 21 June 2026) under the accession number PRJNA1470190.

## Supplementary Materials

The following supporting information can be downloaded at: https://www.mdpi.com/article/10.3390/vetsci13090953/s1, Table S1: RT-qPCR Primers for Differentially Expressed genes; Table S2: Primers for RT-qPCR of miRNA; Table S3: Reverse Transcription System; Table S4: RT-qPCR Reaction System; Table S5: RT-qPCR Reaction Procedure; Table S6: Preparation of Reverse Transcription Systems of miRNA; Table S7: Primers for miRNA Reverse Transcription; Table S8: RT-qPCR Reaction System; Table S9: RT-qPCR Reaction Procedure; Table S10: miRNA Mimic and miRNA Inhibitor Transfection Reagents; Melt Curve Plot.
