# Supplementary material for: Identification of the Potential chi-miR-148a-5p-PTGS2 Regulatory Axis Mediating TNF and IL-17 Signaling Pathways in Caprine Bronchial Epithelial Cells Challenged with Klebsiella Pneumoniae
Source: Vet Sci. 2026 Sep 12;13(9):953. doi: 10.3390/vetsci13090953 (PMC13611970; doi:10.3390/vetsci13090953)
Supplement: Supplementary file 1 [file vetsci-13-00953-s001.zip › supplementary file 1.pdf]

Table S1: RT-qPCR Primers for Differentially Expressed Genes

Gene	Primer sequence (5′→3′)	Product size (bp)
CCL20	F: TGACATCAATGCAGTCGTCTTT<br>R: TTCCTTTCATTCTCGGGCTGT	169
CXCL1	F: CAAGCCGAAGTCATAGCCACT<br>R: TTCCGTTTTCCACCTGGTCA	131
JUN	F: CTTGAAAGCGCAGAACTCGG<br>R: GCGTTAGCATGAGTTGGCAC	119
FOS	F: CTTCAACGCCGACTACGAGG<br>R: TCGGTGCAATAGTCCTGCG	141
TNFAIP3	F: GAGAGAGAACGCCAGCAGAT<br>R: GGCAGTTTCCGTCACCATTT	246
NFKBIZ	F: TGTGAATGCAAAGGCGTACAA<br>R: GCTCGTTCTCCAAGTTCCGA	140
IL1A	F: GCTCGGTTCAGCAAAGAAGTG<br>R: AAGTGGCTCATAGCTTGCGT	153
GAPDH	F: CCTGGAGAAACCTGCCAAGTA<br>R: AAGGTAGAAGAGTGAGTGTCGC	141
IL6	F: TCGCAGGTCTAATAACCACTCCAG<br>R: CAAGGCTTCTCAGGATGATGATAACC	111
CXCL8	F: GGCCACACTGCGAAAATTCA<br>R: CTTGCTTCTCAGCTCTCTTCA	126
TNF	F: CAACGGCGTGGAGCTGAAAG<br>R: TGAAGAGGACCTGCGAGTAGATG	80
PTGS2	F: ATGGGTGTGAAAGGGAGGAAA<br>R: AGTGAAAGCTGGTCCTCGTT	177
TRAF4	F: GCAAGAGAGCGTGTACTGTGA<br>R: GGTACTGGTGACTCTGGATGG	155

Table S2: Primers for RT-qPCR of miRNA

miRNA	Primer sequence (5′→3′)	Product size (bp)
U6	F: GCTTCGGCAGCACATATACTAAAAT<br>R: CGCTTCACGAATTTGCGTGTCAT	89
chi-miR-148a-5p	GSP: GGGGGAAAGTTCTGAGACACT<br>R: GTGCGTGTCGTGGAGTCG	65
miR-1285-z	GSP: CCTGGGCAACATAGCGAGA<br>R: GTGCGTGTCGTGGAGTCG	63
miR-16-x	GSP: GGGGTAGCAGCACGTAAATAT<br>R: GTGCGTGTCGTGGAGTCG	66
miR-6119-x	GSP: GGGGGAGAGGTAAAAAATTGAT<br>R: GTGCGTGTCGTGGAGTCG	66

Table S3: Reverse Transcription System

Component	Volume
5×FastKing-RT SuperMix	4 μL
Total RNA	500 ng
ddH2O	Up to 20 μL

Table S4: RT-qPCR Reaction System

Component	Volume（μL）
2×Premix	10
F	0.4
R	0.4
模板DNA	1.0
ddH2O	Up to 20

Table S5: RT-qPCR Reaction Procedure

Thermal-cycling conditions	Temperature and time		
Thermal-cycling conditions		95℃，30 s	
Denaturation	95℃，5 s		40 cycles
Annealing and extension	60℃，30 s		
Melting curve		95℃，15 s<br>60℃，60 s<br>95℃，1 s	

Table S6: Preparation of Reverse Transcription Systems of miRNA

Component	Volume
dNTP(2.5 mM each)	2.0 μL
10×RT Buffer	2.0 μL
RT-specific primer (1 μM)	0.3 μL
Total RNA	1 μg
MMLV reverse transcriptase (200 U/μL)	0.2 μL
RNase inhibitor (40 U/μL)	0.3 μL
RNase-free water	Up to 20 μL

Table S7: Primers for miRNA Reverse Transcription

miRNA	Primer sequence (5′→3′)
U6	CGCTTCACGAATTTGCGTGTCAT
chi-miR-148a-5p	GTCGTATCCAGTGCGTGTCGTGGAGTCGGCAATTGCACTGGATACGACAGTCGG

miR-1285-z	GTCGTATCCAGTGCGTGTCGTGGAGTCGGCAATTGCACTGGATACGACACAGGG
miR-16-x	GTCGTATCCAGTGCGTGTCGTGGAGTCGGCAATTGCACTGGATACGACATCCCCA
miR-6119-x	GTCGTATCCAGTGCGTGTCGTGGAGTCGGCAATTGCACTGGATACGACAGTCAA

Note: chi-miR-148a-5p is a mature caprine miRNA officially annotated in the miRBase database. In contrast, miR-16-x, miR-6119-x, and miR-1285-z are conserved homologous miRNAs identified through cross-species alignment. The suffixes -x and -z are custom designations generated by the sequencing analysis pipeline and are not official identifiers from miRBase.

Table S8: RT-qPCR Reaction System

Component	Volume（μL）
2×Master Mix	5
10 μM of PCR-specific primer F	0.4
10 μM of the PCR-specific primer R	0.4
cDNA	2.0
ddH2O	Up to 10

Table S9: RT-qPCR Reaction Procedure

Thermal-cycling conditions	Temperature and time	
Thermal-cycling conditions	95℃，10 min	
Denaturation	95℃，10 s	40 cycles
Annealing and extension	60℃，60 s	
Melting curve	95℃，10 s<br>60℃，60 s<br>95℃，15 s	

Table S10: miRNA Mimic and miRNA Inhibitor Transfection Reagents

	Component	Volume
miRNA mimic transfection reagent solution A	RNAiMAX	3 μL
	Opti-MEM	100 μL
miRNA mimic transfection reagent solution B	miRNA mimic	2 μL
	Opti-MEM	100 μL
miRNA inhibi tortransfection reagent solution A	RNAiMAX	3 μL
	Opti-MEM	100 μL
miRNA inhibitortortransfection reagent solution B	miRNA inhibitor	2.5 μL
	Opti-MEM	100 μL
